# Identification and validation of six acute myocardial infarction-associated variants, including a novel prognostic marker for cardiac mortality

**DOI:** 10.3389/fcvm.2023.1226971

**Published:** 2023-07-03

**Authors:** Yeonsu Jeon, Sungwon Jeon, Kyungwhan An, Yeo Jin Kim, Byoung-Chul Kim, Hyojung Ryu, Whan-Hyuk Choi, HyunJoo Choi, Weon Kim, Sang Yeub Lee, Jang-Whan Bae, Jin-Yong Hwang, Min Gyu Kang, Seolbin An, Yeonkyung Kim, Younghui Kang, Byung Chul Kim, Jong Bhak, Eun-Seok Shin

**Affiliations:** ^1^ Korean Genomics Center (KOGIC), Ulsan National Institute of Science and Technology (UNIST), Ulsan, Republic of Korea; ^2^ Clinomics Inc., Ulsan, Republic of Korea; ^3^ Department of Biomedical Engineering, College of Information-Bio Convergence Engineering, Ulsan National Institute of Science and Technology (UNIST), Ulsan, Republic of Korea; ^4^ Department of Mathematics, Kangwon National University, ChunCheon, Republic of Korea; ^5^ Division of Cardiology, Department of Internal Medicine, Kyung Hee University Hospital, Kyung Hee University, Seoul, Republic of Korea; ^6^ Division of Cardiology, Department of Internal Medicine, Chung-Ang University College of Medicine, Chung-Ang University Gwangmyeong Hospital, Gwangmyeong, Republic of Korea; ^7^ Department of Internal Medicine, Chungbuk National University Hospital, College of Medicine, Chungbuk National University, Cheongju, Republic of Korea; ^8^ Department of Internal Medicine, Gyeongsang National University School of Medicine and Gyeongsang National University Hospital, Jinju, Republic of Korea; ^9^ Personal Genomics Institute (PGI), Genome Research Foundation (GRF), Osong, Republic of Korea; ^10^ Department of Cardiology, Ulsan University Hospital, University of Ulsan College of Medicine, Ulsan, Republic of Korea

**Keywords:** acute myocardial infarction, genome-wide association study, cardiac mortality, genetic marker, prognostic marker

## Abstract

**Background:**

Acute myocardial infarction (AMI) is one of the leading causes of death worldwide, and approximately half of AMI-related deaths occur before the affected individual reaches the hospital. The present study aimed to identify and validate genetic variants associated with AMI and their role as prognostic markers.

**Materials and methods:**

We conducted a replication study of 29 previously identified novel loci containing 85 genetic variants associated with early-onset AMI using a new independent set of 2,920 Koreans [88 patients with early- and 1,085 patients with late-onset AMI, who underwent percutaneous coronary intervention (PCI), and 1,747 healthy controls].

**Results:**

Of the 85 previously reported early-onset variants, six were confirmed in our genome-wide association study with a false discovery rate of less than 0.05. Notably, rs12639023, a cis-eQTL located in the intergenic region between *LINC02005* and *CNTN3*, significantly increased longitudinal cardiac mortality and recurrent AMI. *CNTN3* is known to play a role in altering vascular permeability. Another variant, rs78631167, located upstream of *PLAUR* and known to function in fibrinolysis, was moderately replicated in this study. By surveying the nearby genomic region around rs78631167, we identified a significant novel locus (rs8109584) located 13 bp downstream of rs78631167. The present study showed that six of the early-onset variants of AMI are applicable to both early- and late-onset cases.

**Conclusion:**

Our results confirm markers that can potentially be utilized to predict, screen, prevent, and treat candidate patients with AMI and highlight the potential of rs12639023 as a prognostic marker for cardiac mortality in AMI.

## Introduction

Acute myocardial infarction (AMI) is a leading cause of death worldwide, with more than one million deaths per year (1). Elucidating the genetic factors underlying AMI is complex, because environmental factors complicate the etiology of the disease (2). In our previous genome-wide association study (GWAS) (3), we had identified 29 novel genetic loci containing 85 suggestive variants of AMI by targeting 596 patients with early-onset AMI with a high genetic predisposition (4). This study provided evidence to support a genetic association between early-onset AMI and four biochemical pathways: thrombosis, fibrinolysis, inflammation, and lipid metabolism. Our findings showed that an imbalance between thrombosis and fibrinolysis, a conflicting mechanism, may cause AMI. However, it was necessary to validate the replicability of the variants in a new, independent cohort, mainly in the ≥65 years of age cohort, which accounts for most patients with AMI.

Here, we present a replication study of a GWAS using 1,173 new patients with AMI who were eventually treated with percutaneous coronary intervention (PCI) and 1,747 healthy controls from the Korean Genome Project (5). Moreover, we screened for AMI-related variants to determine their association with cardiac mortality and recurrent AMI.

## Materials and methods

### Data sources and study population

A total of 1,173 patients with AMI from the Chungbuk National University Hospital in Korea were enrolled, and 1,747 healthy individuals from the Korean Genome Project (KGP) were selected as controls (5, 6). Patients with AMI were hospitalized with a diagnosis of ST-segment elevation myocardial infarction or non-ST-segment elevation myocardial infarction caused by atherothrombotic occlusive lesions treated with PCI (3, 7). Of the 1,173 patients with AMI, 88 and 1,085 were early-onset and late-onset patients, respectively. Population-based control individuals were obtained from the KGP (5, 6). The KGP is the largest Korean Genome Project and currently includes approximately 10,000 human genomes sequenced in Korea. Information regarding the KGP data can be found on the Korean Genome Project webpage (http://koreangenome.org). The genomes of these 1,173 patients were compared with those of 1,747 control subjects in Korea. Written informed consent was obtained from all participants in this study. Sample collection and sequencing were approved by the Institutional Review Board (IRB) of the Ulsan National Institute of Science and Technology (UNISTIRB-15-19-A). Analyses were performed using Python version 3.7.7 and R version 3.5.0.

### Whole-genome sequencing by MGI T7 sequencer

WGS was performed using the MGI T7 platform, and clinical information from the KGP was matched. A total of 2,920 case and control samples were sequenced for this study in 2020. We filtered and finalized samples according to the following criteria: (i) Case: patients with AMI undergoing PCI surgery and (ii) control: healthy participants without AMI history or stent surgery. In total, 1,129 cases and 1,636 control samples were analyzed for further analysis (Table 1). Genomic DNA was isolated from the blood-containing plates using a DNeasy Blood & Tissue kit (Qiagen, Germany) according to the manufacturer's protocol. The extracted DNA was quantified using a Quant-iT BR Assay Kit (Invitrogen, USA). Each gDNA sample (200 ng) was used to construct a genomic DNA library using the MGIEasy FS DNA Library Prep Set (MGI, Shenhzen, China) according to the manufacturer's instructions. DNA was fragmented by enzymatic fragmentation using magnetic beads. DNA end-repair and adapter ligation were conducted using the MGIEasy DNA Adapters-96 Kit (MGI, Shenzhen, China). The products were run on the 4150 TapeStation (Agilent, Santa Clara, CA, USA), using the Agilent D1000 ScreenTape (Agilent, Santa Clara, CA, USA) to assess the size distribution of the libraries. They were quantified using a Quanti-iT HS Assay Kit (Invitrogen, USA). The PCR products (40 fmol) were circularized and amplified using rolling-circle amplification to generate DNA nanoball-based libraries, which were loaded onto a DNBSEQ-T7RS Sequencing flow cell (MGI, Shenzhen, China) with a DNBSEQ-T7RS High-throughput Sequencing Kit (MGI, Shenzhen, China). The library was run on a DNBSEQ-T7RS (MGI, Shenzhen, China) platform at paired-end 150 bp reads. The quality of bases in the reads was checked using FastQC (ver. 0.11.5; www.bioinformatics.babraham.ac.uk/projects/fastqc/) software. Additional details regarding the genomic variant identification are provided in the Supplementary Methods section of the online Supplementary Information.

**Table 1:** Baseline characteristics in this study.

	Discovery dataset		Replication dataset	
	Early-onset AMI (*N* = 596)	Control (*N* = 636)	AMI (*N* = 1,129)	Control (*N* = 1,636)
Male, *n* (%)	486 (81.5)	328 (51.0)	861 (76.3)	718 (43.9)
Age, median (*Q*1–*Q*3)	46 (42.0–48.0)	44 (29.5–57.0)	65 (58.0–75.0)	45 (32.0–55.0)
Body mass index, mean ± SD	25.5 ± 3.9	24.0 ± 3.4	30.0 ± 109.7	23.7 ± 3.5
Hypertension, *n* (%)	188 (31.5)	90 (14.0)	576 (51)	276 (16.9)
Diabetes mellitus, *n* (%)	118 (19.8)	33 (5.1)	341 (30.2)	112 (6.8)
Current smoking, *n* (%)	360 (60.4)	82 (12.8)	430 (38.1)	170 (10.4)
Lipid levels, mg/dl				
Total cholesterol, mean ± SD	202.7 ± 49.2	179.6 ± 34.3	177.4 ± 47.3	198.6 ± 38.0
LDL cholesterol, mean ± SD	129.1 ± 43.4	115.8 ± 33.0	110.2 ± 39.2	118.1 ± 34.8
HDL cholesterol, mean ± SD	43.9 ± 13.0	57.4 ± 13.9	43.6 ± 12.2	56.8 ± 14.2
Triglycerides, mean ± SD	209.1 ± 185.1	115.8 ± 77.0	144.6 ± 113.6	118.4 ± 73.4

AMI, acute myocardial infarction; LDL, low-density lipoprotein; HDL, high-density lipoprotein.

Values are mean ± SD, median (interquartile range, 25th–75th), or *n* (%).

### Genome-wide association study (GWAS)

GWAS was performed using logistic regression with an additive genetic model using PLINK (ver. 1.9b) (8). A total of 3,484 SNPs and indels, which are near the previous genetic variants associated with early-onset AMI, were tested for replication. Sex and the top ten principal components were included as covariates in the model. The statistically significant *P*-value threshold was determined to be 5.88 × 10^−4^ which is the same as 0.05 FDR value with Bonferroni correction. We assigned significantly replicated variants using the following criteria: (i) variants whose *P*-value was over the significance threshold, and (ii) variants that had a consistent direction of effect in both GWAS results between the discovery and replication datasets.

### Survival analysis of genetic effect on cardiac mortality and recurrent AMI

Survival analysis was performed using the Cox multivariate regression model to estimate the risk of composite outcomes of cardiac mortality and recurrent AMI among patients with AMI by genotype. The survival package in R software (ver. 4.2.2) was used for the analysis (9). Age and sex were also included as covariates in the model. The follow-up period was restricted to 2,000 days (5.5 years). Wild-type genotype carriers were assigned a value of 0, heterozygous genotype carriers a value of 1, and homozygous carriers a value of 2 (10). The Kaplan–Meier curve was used to visualize genetic effects on cardiac mortality and recurrent AMI.

### Quantitative trait loci (QTL) mapping

QTL analysis was performed using an in-house Python script (ver. 3.7.7). Significant variants from the GWAS were queried for their dose-dependent genetic influence on various molecular phenotypes previously reported in the publicly available QTLbase database (ver. 2.0; http://www.mulinlab.org/qtlbase) (11). QTLbase delivers 22 phenotypes ranging from apaQTL (alternative polyadenylation QTL), eQTL (expression QTL), and hQTL (histone QTL) to stQTL (mRNA stability QTL).

## Results

### Replication of genetic variants associated with acute myocardial infarction

**Figure 1:**
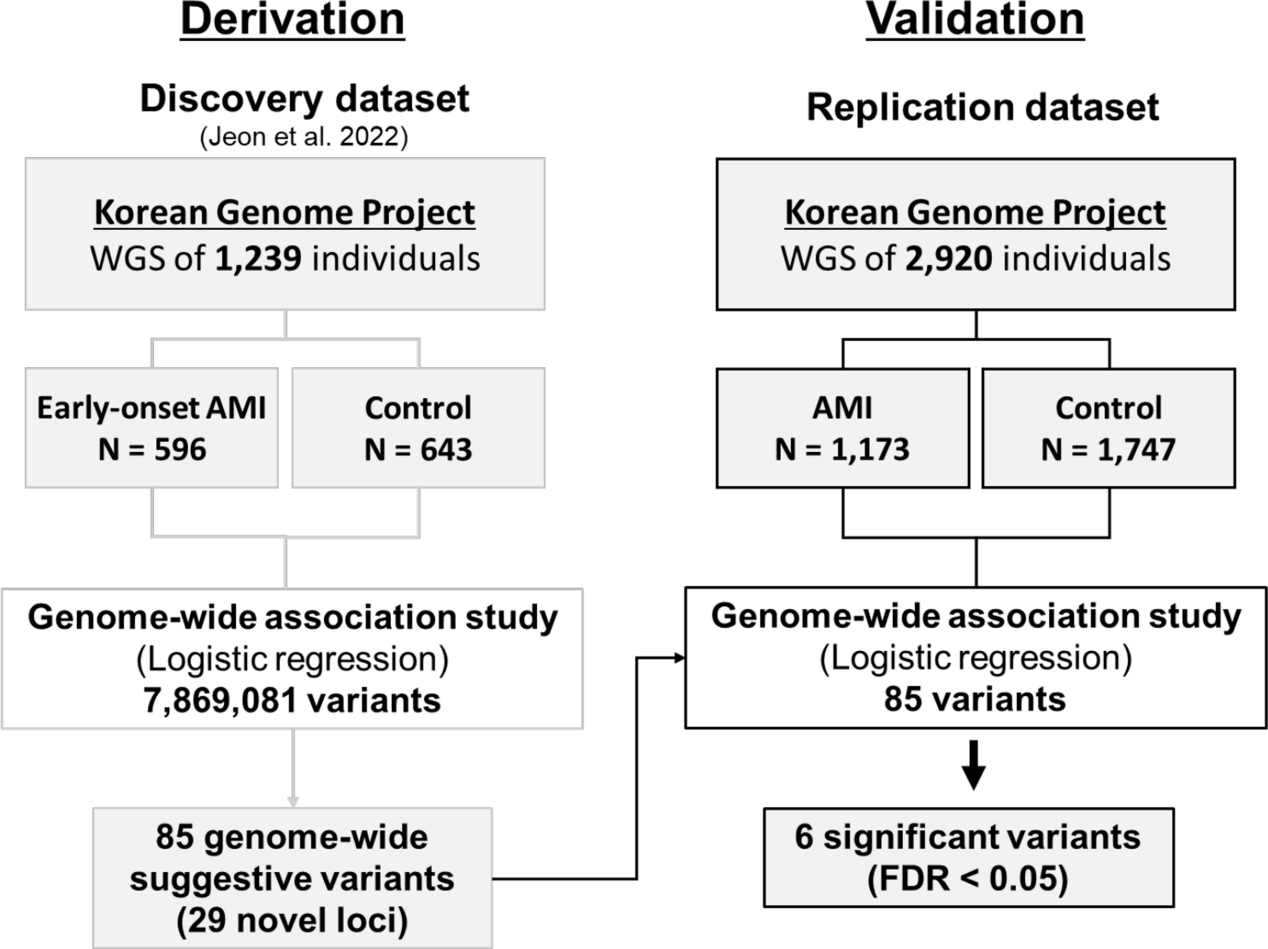
Validation process workflow for genetic variants associated with acute myocardial infarction (AMI).

We found that six variants out of the 85 suggestive early-onset markers were significantly replicated in the AMI group using GWAS over a statistically significant false discovery rate (FDR) threshold (FDR < 0.05; *P* < 5.88 × 10^−4^) (Figure 1, Table 2, Supplementary Table S1, and Supplementary Figure S1). Two intergenic variants (rs12639023 and rs12639020), located near *LINC02005* and *CNTN3* genes, were significantly associated with AMI (OR = 1.432, *P* = 2.17 × 10^−8^ for rs12639023 and OR = 1.267, *P* = 4.05 × 10^−4^ for rs12639020). Another variant, rs12921822 (OR = 1.388, *P* = 2.77 × 10^−4^), located in an intron of RNA binding fox-1 homolog 1 (*RBFOX1*), was also replicated. *RBFOX1* is known to be involved in cardiomyopathy (12). Furthermore, rs1560389462, which is an in-frame deletion in Mucin 4 (*MUC4*), was significantly associated with AMI (OR = 0.093, *P* = 7.60 × 10^−5^). *MUC4* was reported to contribute to cancer progression by suppressing apoptosis and prompting tumor cell proliferation (13). Other two significant variants were located in intergenic regions of *FRG1CP*-*FRG1DP* and *MIR1263*-*LINC01324* (OR = 5.126, *P* = 4.66 × 10^−23^ and OR = 0.400, *P* = 1.13 × 10^−9^) exerting risk and protective effect on the occurrence of AMI, respectively.

**Table 2:** Results of the genome-wide association studies (GWAS) for the replication of genetic variants associated with early-onset AMI.

Gene	Chromosome	Position	rsID	REF	ALT	Effect allele	Discovery (Early-onset AMI)		Replication (AMI)		Survival analysis		
							Odds ratio	*P*	Odds ratio	*P*	HR	95% CI	*P*
*FRG1CP-FRG1DP*	20	28,772,995	rs1277393322	G	A	A	5.713	3.36 × 10^−18^	5.126	4.66 × 10^−23^	0.633	0.271–1.48	0.291
*MIR1263-LINC01324*	3	164,704,630	rs1351282285	TTAAATG	T	T	0.239	5.67 × 10^−10^	0.400	1.13 × 10^−09^	NA	NA	NA
*LINC02005-CNTN3*	3	73,919,636	rs12639023	C	T	C	1.722	2.19 × 10^−06^	1.432	2.17 × 10^−08^	1.371	1.001–1.877	0.049
*MUC4*	3	195,788,126	rs1560389462	GGGTGGTGTGACC TGTGGATACTGAGG AAGTGTCGGTGACA GGAAGAGAGGTGG CGTGACCTGTGGAT GCTGAGGAAGTGTC GGTGACAGGAAGAG GGGTGGTGTGACCT GTGGATACTGAGGA AGTGTCGGTGACAG GAAGAGA	G	G	0.256	1.31 × 10^−08^	0.093	7.60 × 10^−05^	NA	NA	NA
*RBFOX1*	16	7,002,773	rs12921822	C	T	C	1.601	9.05 × 10^−06^	1.388	2.77 × 10^−04^	0.982	0.645–1.495	0.931
*LINC02005-CNTN3*	3	73,919,588	rs12639020	C	T	C	1.722	2.19 × 10^−06^	1.267	4.05 × 10^−04^	1.302	0.939–1.805	0.114
*PLAUR-IRGC*	19	43,676,115	rs78631167	T	C	C	0.473	2.93 × 10^−06^	0.691	1.58 × 10^−03^	1.063	0.525–2.152	0.865
*PLAUR-IRGC*	19	43,676,102	rs8109584	G	A	A	0.981	0.907	0.3531	1.43 × 10^−05^	1.136	0.55–2.347	0.731

In addition, a variant related to fibrinolysis, rs78631167, located upstream of plasminogen activator urokinase receptor (*PLAUR*) was moderately significant in this study (OR = 0.691, *P* = 1.58 × 10^−3^). Although rs78631167 did not show a strong association in the new cohort, rs8109584 showed a significant association with AMI (*P* = 1.43 × 10^−5^), which is 13 bp away from rs78631167, indicating that the genetic block where these variants lie may have a relationship with AMI. *PLAUR* encodes CD87, which converts plasminogen to plasmin, resulting in clot lysis and plaque healing (3, 14).

### Quantitative trait locus associated with acute myocardial infarction

Four significantly replicated variants for AMI and two additional variants upstream of *PLAUR* were found to have at least one quantitative trait locus (QTL), such as eQTL (expression QTL), mQTL (methylation QTL), and tuQTL (transcript usage QTL), from the QTLbase (ver. 2.0), which accumulates information about molecular phenotypes and their QTLs to improve the biological interpretability of genetic markers (Table 3 and Supplementary Table S2). Notably, we found evidence that rs12639020 and rs12639023, which are intergenic variants residing between *LINC02005* and *CNTN3*, may alter the expression and transcript usage of the *CNTN3* gene itself (*P* = 1.54 ×* *10^−4^ in rs12639020 and *P* = 2.93 ×* *10^−3^ in rs12639023; Supplementary Table S2) and its neighboring gene, *PDZRN3* (*P* = 1.12 ×* *10^−4^ in rs12639020 and rs12639023; Table 3). Both *CNTN3* and *PDZRN3* are involved in the regulation of vascular morphology and permeability (15–19). Moreover, the variant at rs12921822 was shown to affect the expression of a novel gene with an unknown function referred to as *AC009135.1* (*P* = 9.08 × 10^−3^; Supplementary Table S2), while also influencing the methylation status of the *RBFOX1* itself (*P* = 2.43 × 10^−18^; Table 3). *RBFOX1* has recurrently proven its relationship with cardiomyopathy (12).

**Table 3:** Representative quantitative trait locus (QTL) results of the replicated variants for AMI by QTLbase.

QTL type	rsID	Gene	Tissue	Affected gene	*P*-value
eQTL	rs12639023	*LINC02005-CNTN3*	Blood-macrophage	*PDZRN3*	1.12 × 10^−04^
eQTL	rs12639020	*LINC02005-CNTN3*	Blood-macrophage	*PDZRN3*	1.12 × 10^−04^
eQTL	rs1560389462	*MUC4*	Artery-tibial	*SMBD1P*	4.18 × 10^−06^
eQTL	rs78631167	*PLAUR-IRGC*	Blood-macrophage	*ZNF235*	6.64 × 10^−05^
eQTL	rs8109584	*PLAUR-IRGC*	Blood	*PLAUR*	6.39 × 10^−13^
eQTL	rs12921822	*RBFOX1*	Brain-prefrontal cortex	*AC009135.1*	9.08 × 10^−03^
hQTL	rs8109584	*PLAUR-IRGC*	Blood-neutrophils CD16+	*PLAUR*	2.62 × 10^−03^
m6AQTL	rs78631167	*PLAUR-IRGC*	Lymphocyte	*IRGQ*	4.06 × 10^−02^
mQTL	rs12639023	*LINC02005-CNTN3*	Blood	*AKR1B1P2*	1.21 × 10^−83^
mQTL	rs8109584	*PLAUR-IRGC*	Blood-monocytes CD14+	*PLAUR*	7.99 × 10^−11^
mQTL	rs12921822	*RBFOX1*	Blood	*RBFOX1*	2.43 × 10^−18^
tuQTL	rs12639023	*LINC02005-CNTN3*	Stem cell-iPSC	*PDZRN3*	7.78 × 10^−03^
tuQTL	rs12639020	*LINC02005-CNTN3*	Stem cell-iPSC	*PDZRN3*	7.78 × 10^−03^
tuQTL	rs8109584	*PLAUR-IRGC*	Stem cell-iPSC	*PLAUR*	1.98 × 10^−03^

Representative QTL results show the QTL with the lowest *P*-value per genetic variant and QTL type.

### Genetic effects on cardiac mortality and recurrent acute myocardial infarction

**Figure 2:**
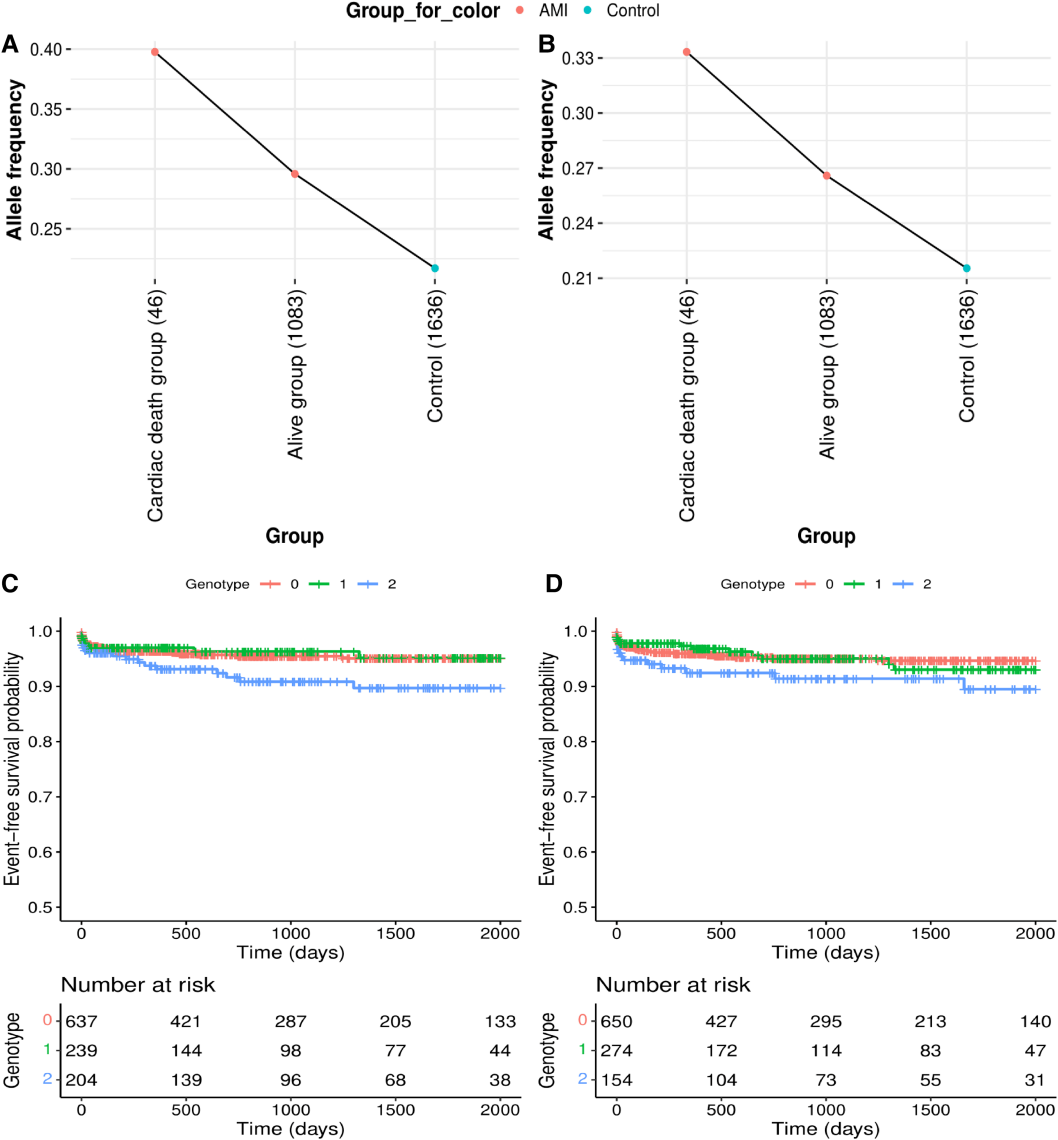
Genetic effects on cardiac mortality and recurrent acute myocardial infarction (AMI). (**A,B**) Comparison of allele frequencies among cardiac death, alive and control groups in (**A**) rs12639023, *LINC02005*-*CNTN3* and (**B**) rs12639020, *LINC02005*-*CNTN3*. (**C,D**) Kaplan–Meier curves for two variants with a positive genetic effect for cardiac mortality and recurrent AMI. (**C**) Survival curve by rs12639023 genotype. (**D**) Survival curve by rs12639020 genotype. *X*-axis denotes days from enrollment to death or last follow-up. *Y*-axis denotes event-free survival probability. Event means cardiac mortality and recurrent AMI.

Of the six significantly replicated variants for AMI and two additional variants upstream of *PLAUR*, seven variants had greater allele frequency differences between AMI patients with cardiac death and controls than between living patients and controls (Figures 2A,B, and Supplementary Table S3). Rs12639023, located near the *LINC02005* and the *CNTN3*, showed the largest difference in allele frequency between the cardiac death and the living groups among the patients with AMI (Figure 2A; allele frequency in the cardiac death group = 0.3977, allele frequency in the living group = 0.2958, and allele frequency in the control group = 0.2171).

Furthermore, the two variants were found to have enhancing effects [Hazard Ratio (HR) > 1] on longitudinal cardiac mortality and recurrent AMI when the survival associations between the variants and cardiac mortality were analyzed using longitudinal follow-up data after PCI in individuals with AMI (Table 2, Figures 2C,D, and Supplementary Figure S2). Among these, rs12639023 was the only variant with a significant enhancing effect (HR = 1.371, *P* = 0.049) on longitudinal cardiac mortality and recurrent AMI (Figure 2C).

## Discussion

This study aimed to identify replicated genetic variants of AMI from 85 previously reported variants associated with early-onset AMI using a new set of 2,920 Koreans. The new independent replication dataset consisted mainly of patients with late-onset AMI. We hypothesized that there are markers that are applicable to both stages or types of AMI. Nonetheless, our sample size including 1,747 healthy controls is still relatively small.

Among the variants related to the four major mechanisms previously mentioned for early-onset AMI (3), a fibrinolysis-related variant was moderately replicated in this study, whereas variants related to thrombosis, inflammation, and lipid metabolism were not significantly replicated. This may be because the replication dataset was mainly composed of patients with late-onset AMI, who were probably more affected by environmental risk factors than by genetic factors. We anticipate that the variants related to the remaining major mechanisms may be significantly replicated if the dataset includes a larger number of patients with early-onset AMI with a high genetic predisposition in the replication dataset. Nevertheless, a variant related to fibrinolysis, rs78631167, located upstream of *PLAUR*, had a modest effect (*P* = 1.58 × 10^−3^). Moreover, rs8109584, located 13 bp downstream of rs78631167, was significantly associated with AMI (*P* = 1.43 × 10^−5^), suggesting that the genetic block located upstream of *PLAUR* is significantly associated with AMI.

The results of the present study propose that vascular permeability could serve as a crucial mechanism for predicting the occurrence and prognosis of AMI. Of the significantly replicated variants, rs12639023, located near *LINC02005* and *CNTN3*, showed the highest enhancing effect on longitudinal cardiac mortality and recurrent AMI expression. It is also a cis-eQTL that increases the expression of *CNTN3* encoding contactin 3 (Supplementary Table S2). Notably, contactin 3 is an activator of Arf6 that has been reported to affect inflammation-induced vascular permeability (15, 16). Elevated vascular permeability causes trackable macromolecules to extravasate, thereby threatening endothelial integrity and dysfunction in cardiovascular disease (19). However, the role of vascular permeability in AMI has not been sufficiently focused on. Phinikaridou et al. demonstrated that vascular permeability is elevated in atherosclerotic vessels compared to stable vessels, and it is higher in rupture-prone than in stable atherosclerotic lesions in a rabbit model (20, 21). Nonetheless, there was a paucity of human studies investigating the association between vascular permeability and AMI in patients, who experience atherosclerotic plaque rupture as a major cause of AMI. The present study suggests that increased vascular permeability could be a potential target as a major pathogenic mechanism contributing to plaque development and rupture, which represent primary steps in the development of AMI. Furthermore, our longitudinal result implies that patients with increased vascular permeability, even after PCI, may face a higher risk of plaque development and rupture, indicating a poor prognosis.

The two key strengths of our study include clearly defined sample collection and replication of genetic markers using thousands of samples in an independent cohort. While most studies have used patients with AMI with heterogeneous phenotypes (22, 23), the present study was designed to include only those patients with AMI who underwent PCI for atherothrombotic occlusive lesions to prevent misclassification of the AMI phenotype. Therefore, AMI samples with non-obstructive and non-atherothrombotic causes, such as spontaneous coronary artery dissection, spasm, and thromboembolism, were excluded. Strict sample filtering allowed us to identify and validate genetic factors related to atherothrombosis in AMI, which correspond to most AMI phenotypes. In addition, this study confirmed the replication of potential genetic markers associated with AMI using 2,920 individuals in an independent cohort. This provides evidence that these markers can be used to predict and diagnose AMI.

Our study has two limitations. First, this study was conducted in patients with AMI of Korean ancestry. These results should be validated in populations of multiple races. Second, this study confirmed the replication of AMI-associated variants in a small sample size of 2,920 individuals. While significant variants were observed not in the replication but in the discovery phase, rs8109584, which is 13 bp away from rs78631167, was significantly found in the replication rather than the discovery phase. It is necessary to increase the sample size to ensure validation.

In conclusion, this study confirmed the replication of six genetic variants associated with AMI by using GWAS, QTL mapping, and survival analyses. Our findings highlight the potential of rs12639023 as a prognostic marker for cardiac mortality in AMI. These results provide insights into the latent etiology of AMI. The replicated genetic variants are potential biomarkers for the prediction, prevention, prognosis, and personalized treatment of AMI in individuals who carry the variant. They could be clinically utilized in routine health check-ups to assess the risk of AMI by a genetic risk score derived from these variants, enabling the early prediction of individuals with a higher genetic predisposition for AMI.

## Data Availability

Full summary statistics relating to the GWAS analysis generated during the current study is available on the Korean Genome Project website (http://koreangenome.org/Cardiomics). All other relevant data are available upon request from the authors.
