# Supplementary material for: Identification and validation of six acute myocardial infarction-associated variants, including a novel prognostic marker for cardiac mortality
Source: Front Cardiovasc Med. 2023 Jul 3;10:1226971. doi: 10.3389/fcvm.2023.1226971 (PMC10350496; doi:10.3389/fcvm.2023.1226971)
Supplement: Supplementary file 1 [file Datasheet1.pdf]

Supplementary Information

Identification and Validation of Six Acute Myocardial Infarction-associated Variants, including a Novel Prognostic Marker for Cardiac Mortality

SUPPLEMENTARY METHODS

Genomic variants identification

We trimmed the adapter contamination of the sequencing reads using Cutadapt (ver. 1.9.1) with a forward adapter (′GATCGGAAGAGCACACGTCTGAACTCCAGTCAC′) and reverse adapter (′GATCGGAAGAGCGTCGTGTAGGGAAAGAGTGT′) and with a minimum read length of 50 bp after trimming (1). Then, the trimmed reads were mapped to the human genome reference, hg38 using BWA-MEM (ver. 0.7.16a) with the “-M” option and alt-aware mode (2). We sorted the mapped BAM files by coordination using Picard (“Picard Toolkit.” 2019. Broad Institute, GitHub Repository. https://broadinstitute.github.io/picard/; Broad Institute) (ver. 2. 20. 3) with the Sortsam module. Duplicated reads were marked using Picard (ver. 2.20.3) with the MarkDuplicates module. We recalibrated the mapping quality using BaseRecalibrator tool in the Genome Analysis Tool Kit (GATK) (ver. 4.1.3) (3). gVCF files of the individuals were generated by HaplotypeCaller in GATK with the “--genotyping-mode DISCOVERY -stand-call-conf 30 -ERC GVCF” option (3). We defined the target genotyping sites as upstream and downstream 20 bases of the 85 previously identified variants associated with early-onset AMI (4). We genotyped individuals genotypes on the target sites using GenotypeGVCFs in GATK. The variants were annotated using the Ensembl VEP (ver. 92.1) and ANNOVAR (5, 6).

SUPPLEMENTARY FIGURES

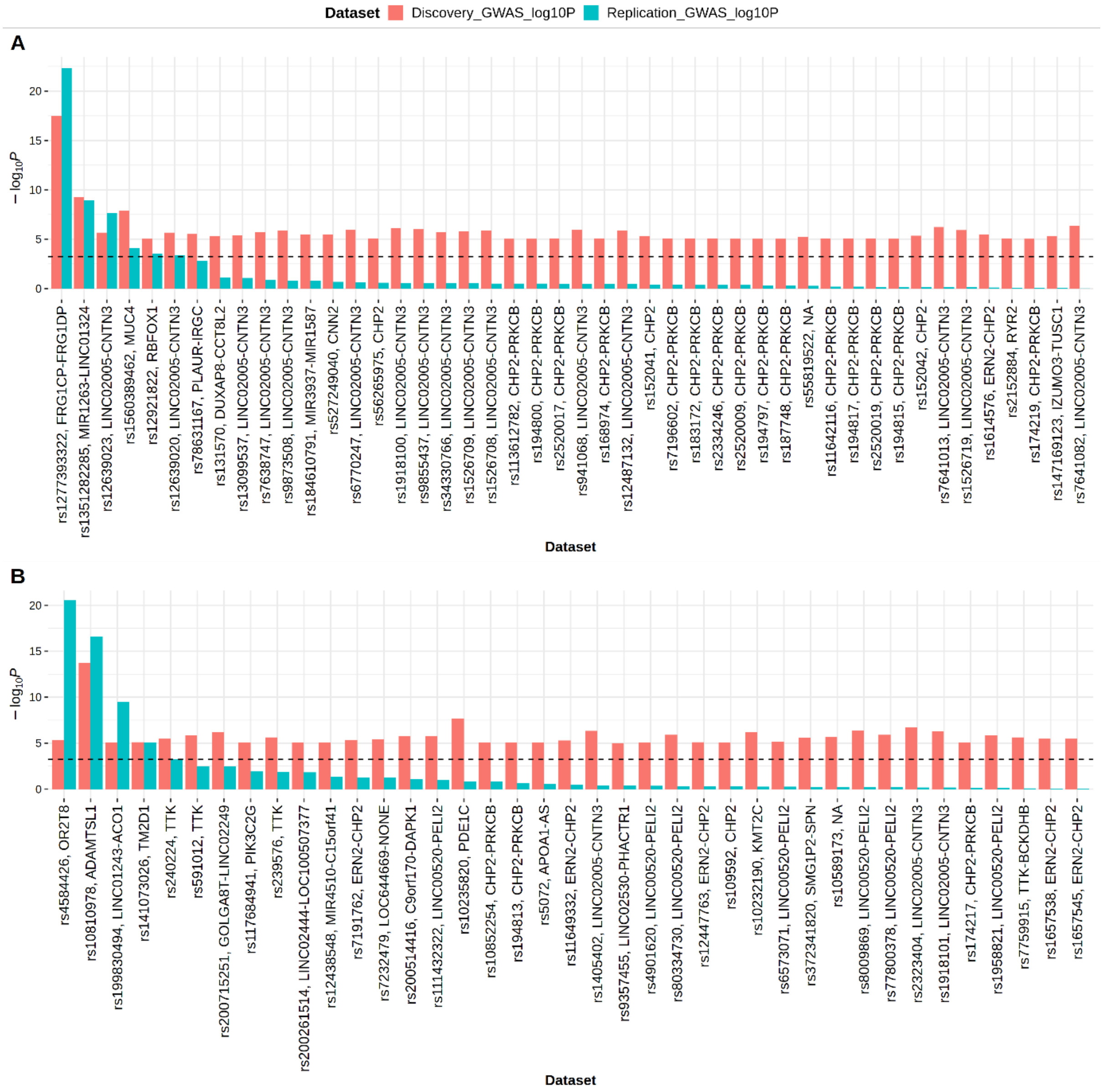

Supplementary Figure S1. Comparison of *p*-values between discovery and replication GWAS

X-axis indicates (A) 46 variants with consistent direction of effect and (B) 39 variants with inconsistent direction of effect between discovery and replication GWAS. Y-axis indicates -$\log_{10}$ *P*-value. The dotted line indicates a significant threshold (FDR < 0.05; $P < 5.88 \times 10^{-4}$). Red and Cyan indicate discovery and replication dataset, respectively.

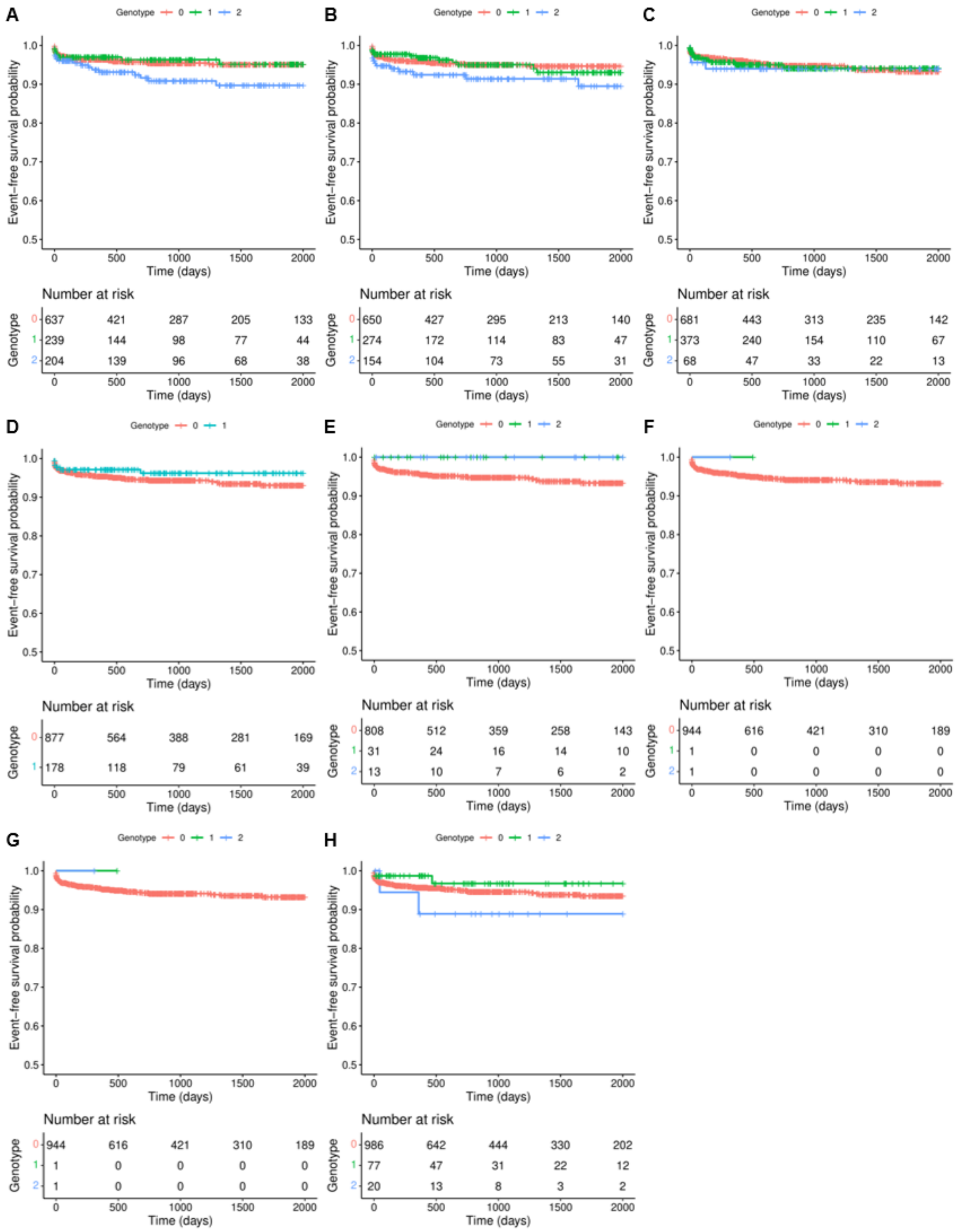

Supplementary Figure S2. Survival curve of six significant replicated variants and two variants near *PLAUR*

Survival curve by (A) rs12639023, (B) rs12639020, (C) rs12921822, (D) rs1277393322, (E) rs1351282285, (F) rs1560389462, (G) rs78631167, (H) rs8109584 genotypes. X-axis denotes days from enrollment to death or last follow-up. Y-axis denotes event-free survival probability. Event means cardiac mortality and future spontaneous AMI.

SUPPLEMENTARY TABLES

Supplementary Table S1. Results of the genome-wide association studies (GWAS) for 85 previously identified variants for early-onset AMI

Gene	Chromosome	Position	rsID	REF	ALT	Effect allele	Discovery (Early-onset AMI)		Replication (AMI)	
							Odds ratio	*P*	Odds ratio	*P*
FRG1CP-FRG1DP	20	28,772,995	rs1277393322	G	A	A	5.7130	3.36E-18	5.1260	4.66E-23
ADAMTSL1	9	18,523,845	rs10810978	C	A	C	0.4509	1.94E-14	1.7538	2.49E-17
MIR1263-LINC01324	3	164,704,630	rs13512822 85	TTAAATG	T	T	0.2394	5.67E-10	0.4002	1.13E-09
LINC02005-CNTN3	3	73,919,636	rs12639023	C	T	C	1.7220	2.19E-06	1.4320	2.17E-08
PDE1C	7	32,082,944	rs10235820	A	G	A	0.4970	2.21E-08	1.1110	0.1403
LINC02005-CNTN3	3	73,926,364	rs2323404	A	G	A	1.6060	1.98E-07	0.9630	0.6505
MUC4	3	195,788,126	rs1560389462	GGGTGGTGTGACCTGTGGATACTGAGGAAGTGTCGGTGACAGGAAGAGAGGTGGCGTGACCTGTGGATGCTGAGGAAGTGTCGGTGACAGGAAGAGGGGTGGTGTGACCTGTGGATACTGAGGAAGTGTCGGTGACAGGAAGAGA	G	G	0.2562	1.31E-08	0.0926	7.6E-05
LINC00520-PELI2	14	55,900,262	rs8009869	A	G	G	1.6530	4.49E-07	0.9673	0.6314
LINC02005-CNTN3	3	73,921,969	rs1405402	G	A	G	1.5790	4.81E-07	0.9315	0.3927
LINC02005-CNTN3	3	73,927,087	rs1918101	G	T	G	1.5760	5.47E-07	0.9643	0.669
RBFOX1	16	7,002,773	rs12921822	C	T	C	1.6010	9.05E-06	1.3880	0.000277
KMT2C	7	152,231,944	rs10232190	G	A	A	1.9800	6.24E-07	0.9239	0.5254
GOLGA8T-LINC02249	15	30,175,826	rs200715251	A	G	G	1.8400	6.56E-07	0.7268	0.00333
LINC02005-CNTN3	3	73,919,588	rs12639020	C	T	C	1.7220	2.19E-06	1.2670	0.000405
PLAUR-IRGC	19	43,676,115	rs78631167	T	C	C	0.4725	2.93E-06	0.6905	0.001579
DUXAP8-CCT8L2	22	16,400,343	rs131570	T	C	C	0.5444	4.66E-06	0.8512	0.07497
LINC02005-CNTN3	3	73,923,060	rs13099537	T	C	T	1.6960	4.17E-06	1.1290	0.0851
LINC02005-CNTN3	3	73,923,365	rs7638747	A	G	A	1.7280	1.97E-06	1.1140	0.1292

LINC00520-PELI2	14	55,906,845	rs80334730	T	C	C	1.6150	1.22E-06	0.9513	0.4697
LINC00520-PELI2	14	55,907,084	rs77800378	G	A	A	1.6150	1.22E-06	0.9675	0.6329
LINC02005-CNTN3	3	73,925,782	rs9873508	G	A	G	1.7400	1.33E-06	1.1060	0.1574
MIR3937-MIR1587	X	39,788,017	rs184610791	C	G	G	0.2344	3.23E-06	0.7832	0.1595
CNN2	19	1,029,328	rs527249040	G	C	C	0.2916	3.2E-06	0.6926	0.2094
LINC00520-PELI2	14	55,908,821	rs1958821	G	C	C	1.6140	1.44E-06	0.9817	0.7897
TTK	6	80,038,900	rs591012	G	A	G	0.5831	1.47E-06	1.4180	0.003043
CHP2-PRKCB	16	23,791,390	rs194813	T	C	C	1.6050	9.12E-06	0.9054	0.239
C9orf170-DAPK1	9	87,414,203	rs2005144166	T	C	C	1.9480	1.78E-06	0.7017	0.08624
LINC00520-PELI2	14	55,905,697	rs111432322	C	T	T	1.6010	1.83E-06	0.8934	0.1046
LINC02005-CNTN3	3	73,927,363	rs6770247	G	A	G	1.7480	1.08E-06	1.0860	0.2471
LINC02005-CNTN3	3	73,927,219	rs1918100	T	A	T	1.7640	7.45E-07	1.0820	0.2719
NA	14	55,905,174	rs10589173	ATATAT	A	A	1.5980	2.02E-06	0.9643	0.6023
LINC02005-CNTN3	3	73,925,188	rs9855437	A	G	A	1.7540	9.6E-07	1.0800	0.2828
LINC02005-CNTN3	3	73,924,589	rs34330766	A	G	A	1.7310	1.88E-06	1.0790	0.2943
TTK	6	80,021,249	rs239576	G	A	G	0.5890	2.41E-06	1.3620	0.01255
TTK-BCKDHB	6	80,062,270	rs7759915	C	T	T	0.5890	2.41E-06	1.0240	0.8446
SMG1P2-SPN	16	29,655,837	rs372341820	G	A	A	0.1804	2.75E-06	1.2340	0.5932
LINC02005-CNTN3	3	73,918,900	rs1526709	C	A	C	1.7360	1.59E-06	1.0780	0.2963
LINC02005-CNTN3	3	73,918,804	rs1526708	G	A	G	1.7440	1.34E-06	1.0770	0.3074
CHP2-PRKCB	16	23,761,303	rs113612782	G	A	A	1.6100	8.92E-06	1.0910	0.3126
CHP2-PRKCB	16	23,763,247	rs194800	T	C	C	1.6110	9.09E-06	1.0900	0.3218
ERN2-CHP2	16	23,747,877	rs1657538	G	A	A	1.6460	3.41E-06	0.9854	0.865
ERN2-CHP2	16	23,748,316	rs1657545	A	G	G	1.6460	3.41E-06	0.9906	0.9121
TTK	6	80,007,571	rs240224	G	T	G	0.5943	3.45E-06	1.5720	0.00057
LOC644669-NONE	18	15,377,667	rs7232479	G	A	A	1.7630	3.82E-06	0.7806	0.05232

CHP2-PRKCB	16	23,784,944	rs2520017	G	A	A	1.6160	7.94E-06	1.0880	0.3327
LINC02005-CNTN3	3	73,925,641	rs941068	C	T	C	1.7480	1.08E-06	1.0720	0.336
CHP2-PRKCB	16	23,789,042	rs168974	T	A	A	1.6160	7.94E-06	1.0850	0.3454
ERN2-CHP2	16	23,741,456	rs7191762	G	A	A	1.6590	4.82E-06	0.8568	0.05206
OR2T8	1	247,921,552	rs4584426	A	G	G	1.5380	4.83E-06	0.3473	2.8E-21
LINC02005-CNTN3	3	73,919,134	rs12487132	A	C	A	1.7470	1.26E-06	1.0700	0.3489
ERN2-CHP2	16	23,748,912	rs11649332	C	T	T	1.6320	4.98E-06	0.8811	0.359
CHP2	16	23,754,034	rs152041	G	C	C	1.6320	4.93E-06	1.0730	0.416
CHP2-PRKCB	16	23,786,294	rs7196602	C	T	T	1.6160	7.94E-06	1.0720	0.4217
LINC00520-PELI2	14	55,904,081	rs6573071	G	A	A	1.5310	6.72E-06	0.9631	0.5619
ERN2-CHP2	16	23,742,273	rs12447763	G	T	T	1.6610	7.6E-06	0.9479	0.5108
CHP2-PRKCB	16	23,789,509	rs183172	T	C	C	1.6160	7.94E-06	1.0690	0.4389
CHP2-PRKCB	16	23,787,611	rs2334246	A	T	T	1.6160	7.94E-06	1.0680	0.441
CHP2-PRKCB	16	23,769,092	rs2520009	C	T	T	1.6100	8.92E-06	1.0670	0.4488
CHP2-PRKCB	16	23,767,186	rs187748	G	A	A	1.6100	8.92E-06	1.0590	0.5074
CHP2	16	23,757,389	rs109592	C	T	T	1.6090	8.63E-06	0.9467	0.5235
NA	16	23,771,655	rs55819522	TAA	T	T	1.6240	6.41E-06	1.0530	0.5488
CHP2-PRKCB	16	23,786,091	rs11642116	A	C	C	1.6160	7.94E-06	1.0390	0.6526
CHP2-PRKCB	16	23,788,500	rs194817	G	A	A	1.6160	7.94E-06	1.0380	0.6654
CHP2-PRKCB	16	23,785,567	rs2520019	T	C	C	1.6160	7.94E-06	1.0320	0.7118
TM2D1	1	61,717,206	rs141073026	A	C	C	1.6730	8.13E-06	0.6923	8.82E-06
LINC02444-LOC100507377	12	73,222,956	rs200261514	G	GTAAT	GTAAT	2.6440	8.25E-06	0.6951	0.01486
APOA1-AS	11	116,836,867	rs5072	A	G	A	1.5450	8.61E-06	0.8938	0.2725
CHP2	16	23,759,927	rs56265975	T	G	G	1.6090	8.63E-06	1.1010	0.2673
CHP2	16	23,754,252	rs152042	A	G	G	1.6350	4.52E-06	1.0300	0.7311
MIR4510-C15orf41	15	36,119,067	rs12438548	G	A	G	0.6094	8.72E-06	1.1620	0.04325
LINC01243-ACO1	9	32,154,607	rs199830494	G	A	G	0.6313	8.77E-06	1.4590	3.32E-10

LINC02005-CNTN3	3	73,923,383	rs7641013	T	C	T	1.5740	5.92E-07	1.0280	0.734
LINC02005-CNTN3	3	73,927,320	rs1526719	A	G	G	1.7450	1.2E-06	1.0220	0.7651
CHP2-PRKCB	16	23,769,518	rs194797	T	C	C	1.6100	8.92E-06	1.0590	0.5031
CHP2-PRKCB	16	23,768,995	rs174217	A	G	G	1.6100	8.92E-06	0.9770	0.7874
ERN2-CHP2	16	23,747,804	rs1614576	A	G	G	1.6460	3.41E-06	1.0200	0.8156
PIK3C2G	12	18,556,791	rs117684941	T	C	C	0.2487	8.95E-06	1.6920	0.01242
LINC00520-PELI2	14	55,901,317	rs4901620	G	A	A	1.5230	8.96E-06	0.9516	0.4424
RYR2	1	237,214,855	rs2152884	G	A	A	1.5300	8.02E-06	1.0180	0.8244
CHP2-PRKCB	16	23,791,599	rs174219	T	C	C	1.6050	9.12E-06	1.0160	0.8549
CHP2-PRKCB	16	23,790,378	rs10852254	T	C	C	1.6050	9.12E-06	0.8829	0.1416
IZUMO3-TUSC1	9	25,189,022	rs147169123	A	AT	A	0.5226	5.14E-06	0.9688	0.8989
CHP2-PRKCB	16	23,790,834	rs194815	A	G	G	1.6050	9.12E-06	1.0310	0.7219
LINC02005-CNTN3	3	73,923,420	rs7641082	T	C	T	1.5850	4.08E-07	1.0010	0.994
LINC02530-PHACTR1	6	12,693,654	rs9357455	C	T	C	0.6658	9.73E-06	1.0890	0.399

Supplementary Table S2. Quantitative trait locus (QTL) results of the replicated variants for AMI by QTLbase

QTL type	rsID	Gene	Tissue	Affected gene	*P*-value
eQTL	rs12639023	*LINC02005-CNTN3*	Blood-Macrophage	*PDZRN3*	0.000112
eQTL	rs12639023	*LINC02005-CNTN3*	Stem cell-iPSC	*PDZRN3*	0.000784
eQTL	rs12639023	*LINC02005-CNTN3*	Stem cell-iPSC	*PDZRN3*	0.00261
eQTL	rs12639023	*LINC02005-CNTN3*	Ovary	*CNTN3*	0.00293
eQTL	rs12639023	*LINC02005-CNTN3*	Blood-Macrophage	*PDZRN3*	0.00472
eQTL	rs12639023	*LINC02005-CNTN3*	Stem cell-iPSC	*PDZRN3*	0.00618
eQTL	rs12639023	*LINC02005-CNTN3*	Blood-Macrophage	*PDZRN3*	0.00766
eQTL	rs12639023	*LINC02005-CNTN3*	Stem cell-iPSC	*PDZRN3*	0.0109
eQTL	rs12639023	*LINC02005-CNTN3*	Stem cell-iPSC	*CNTN3*	0.0127
eQTL	rs12639023	*LINC02005-CNTN3*	Stem cell-iPSC	*PDZRN3*	0.0187
eQTL	rs12639023	*LINC02005-CNTN3*	Stem cell-iPSC	*CNTN3*	0.0347
eQTL	rs12639020	*LINC02005-CNTN3*	Blood-Macrophage	*PDZRN3*	0.000112
eQTL	rs12639020	*LINC02005-CNTN3*	Central Nervous System	*CNTN3*	0.000154
eQTL	rs12639020	*LINC02005-CNTN3*	Stem cell-iPSC	*PDZRN3*	0.000784
eQTL	rs12639020	*LINC02005-CNTN3*	Stem cell-iPSC	*PDZRN3*	0.00261
eQTL	rs12639020	*LINC02005-CNTN3*	Ovary	*CNTN3*	0.00293
eQTL	rs12639020	*LINC02005-CNTN3*	Blood-Macrophage	*PDZRN3*	0.00472
eQTL	rs12639020	*LINC02005-CNTN3*	Stem cell-iPSC	*PDZRN3*	0.00618
eQTL	rs12639020	*LINC02005-CNTN3*	Blood-Macrophage	*PDZRN3*	0.00766
eQTL	rs12639020	*LINC02005-CNTN3*	Stem cell-iPSC	*PDZRN3*	0.0109
eQTL	rs12639020	*LINC02005-CNTN3*	Stem cell-iPSC	*CNTN3*	0.0127
eQTL	rs12639020	*LINC02005-CNTN3*	Stem cell-iPSC	*PDZRN3*	0.0187
eQTL	rs12639020	*LINC02005-CNTN3*	Stem cell-iPSC	*CNTN3*	0.0347
eQTL	rs1560389462	*MUC4*	Artery-Tibial	*SMBD1P*	4.18E-06
eQTL	rs1560389462	*MUC4*	Esophagus	*SMBD1P*	2.38E-05
eQTL	rs1560389462	*MUC4*	Thyroid Gland	*SMBD1P*	4.69E-05

eQTL	rs1560389462	*MUC4*	Central Nervous System	*AC069257.2*	7.92E-05
eQTL	rs78631167	*PLAUR-IRGC*	Blood-Macrophage	*ZNF235*	6.64E-05
eQTL	rs78631167	*PLAUR-IRGC*	Stem cell-iPSC	*AC245748.3*	0.00112
eQTL	rs78631167	*PLAUR-IRGC*	Blood	*CD177*	0.00129
eQTL	rs78631167	*PLAUR-IRGC*	Blood-B cell	*ZNF404*	0.00229
eQTL	rs78631167	*PLAUR-IRGC*	Blood-Monocyte	*ZNF428*	0.00233
eQTL	rs78631167	*PLAUR-IRGC*	Blood-B cell	*ZNF155*	0.00238
eQTL	rs78631167	*PLAUR-IRGC*	Peripheral Nervous System	*ZNF428*	0.00299
eQTL	rs78631167	*PLAUR-IRGC*	Lymphocyte	*ZNF404*	0.00319
eQTL	rs78631167	*PLAUR-IRGC*	Blood-T cell CD8+	*ZNF225*	0.00334
eQTL	rs78631167	*PLAUR-IRGC*	Kidney	*XRCC1*	0.0047
eQTL	rs78631167	*PLAUR-IRGC*	Stem cell-iPSC	*LYPD5*	0.00472
eQTL	rs78631167	*PLAUR-IRGC*	Blood-B cell	*ZNF226*	0.00685
eQTL	rs78631167	*PLAUR-IRGC*	Lymphocyte	*ZNF235*	0.00789
eQTL	rs78631167	*PLAUR-IRGC*	Stem cell-iPSC	*L34079.2*	0.00819
eQTL	rs78631167	*PLAUR-IRGC*	Stem cell-iPSC	*AC005392.2*	0.0087
eQTL	rs78631167	*PLAUR-IRGC*	Blood-Monocyte	*ZNF234*	0.00889
eQTL	rs78631167	*PLAUR-IRGC*	Lymphocyte	*ZNF222*	0.00986
eQTL	rs78631167	*PLAUR-IRGC*	Blood	*SMG9*	0.0118
eQTL	rs78631167	*PLAUR-IRGC*	Blood	*ZNF404*	0.0289
eQTL	rs8109584	*PLAUR-IRGC*	Blood	*PLAUR*	6.39E-13
eQTL	rs8109584	*PLAUR-IRGC*	Blood	*CADM4*	3.36E-05
eQTL	rs8109584	*PLAUR-IRGC*	Blood-T cell CD4+ naive	*ZNF283*	6.56E-05
eQTL	rs8109584	*PLAUR-IRGC*	Kidney	*XRCC1*	0.000286
eQTL	rs8109584	*PLAUR-IRGC*	Blood-T cell CD8+	*ZNF235*	0.000437
eQTL	rs8109584	*PLAUR-IRGC*	Liver	*PSG9*	0.000517
eQTL	rs8109584	*PLAUR-IRGC*	Blood-B cell	*ETHE1*	0.00063
eQTL	rs8109584	*PLAUR-IRGC*	Liver	*ZNF404*	0.000862
eQTL	rs8109584	*PLAUR-IRGC*	Blood-Monocytes CD14+	*ZNF45*	0.000971

eQTL	rs8109584	*PLAUR-IRGC*	Blood-Macrophage	*ZNF155*	0.00133
eQTL	rs8109584	*PLAUR-IRGC*	Blood-T cell CD8+	*ZNF225*	0.00138
eQTL	rs8109584	*PLAUR-IRGC*	Stem cell-iPSC	*SMG9*	0.00154
eQTL	rs8109584	*PLAUR-IRGC*	Blood-Neutrophils CD16+	*AC243964.3*	0.00178
eQTL	rs8109584	*PLAUR-IRGC*	Blood-Monocytes CD14+	*ZNF45*	0.00185
eQTL	rs8109584	*PLAUR-IRGC*	Blood-T cell CD4+	*L34079.3*	0.00226
eQTL	rs8109584	*PLAUR-IRGC*	Blood-Monocyte	*CEACAM19*	0.00243
eQTL	rs8109584	*PLAUR-IRGC*	Blood-Monocyte	*PLAUR*	0.00271
eQTL	rs8109584	*PLAUR-IRGC*	Ovary	*ZNF235*	0.00289
eQTL	rs8109584	*PLAUR-IRGC*	Blood-Neutrophils CD16+	*ZNF230*	0.00325
eQTL	rs8109584	*PLAUR-IRGC*	Blood-Neutrophils CD16+	*XRCC1*	0.00327
eQTL	rs8109584	*PLAUR-IRGC*	Blood-T cell CD4+	*ZNF227*	0.00332
eQTL	rs8109584	*PLAUR-IRGC*	Blood-T cell CD4+	*AC006213.3*	0.00431
eQTL	rs8109584	*PLAUR-IRGC*	Kidney	*PINLYP*	0.00444
eQTL	rs8109584	*PLAUR-IRGC*	Ovary	*ZNF234*	0.0046
eQTL	rs8109584	*PLAUR-IRGC*	Stem cell-iPSC	*ZNF155*	0.00464
eQTL	rs8109584	*PLAUR-IRGC*	Stem cell-iPSC	*LYPD3*	0.00469
eQTL	rs8109584	*PLAUR-IRGC*	Stem cell-iPSC	*ZNF224*	0.00476
eQTL	rs8109584	*PLAUR-IRGC*	Stem cell-iPSC	*PLAUR*	0.00685
eQTL	rs8109584	*PLAUR-IRGC*	Stem cell-iPSC	*IRGQ*	0.00721
eQTL	rs8109584	*PLAUR-IRGC*	Blood-T cell CD4+	*ZNF575*	0.00785
eQTL	rs8109584	*PLAUR-IRGC*	Blood-Neutrophils CD16+	*SMG9*	0.00788
eQTL	rs8109584	*PLAUR-IRGC*	Blood-T cell CD4+	*AC006213.1*	0.00817
eQTL	rs8109584	*PLAUR-IRGC*	Blood-B cell	*ZNF180*	0.00854

eQTL	rs8109584	*PLAUR-IRGC*	Blood-T cell CD8+	*ZNF230*	0.0087
eQTL	rs8109584	*PLAUR-IRGC*	Stem cell-iPSC	*ZNF428*	0.00894
eQTL	rs8109584	*PLAUR-IRGC*	Blood	*ZNF234*	0.00939
eQTL	rs8109584	*PLAUR-IRGC*	Blood	*KCNN4*	0.0131
eQTL	rs8109584	*PLAUR-IRGC*	Stem cell-iPSC	*ZNF404*	0.0135
eQTL	rs8109584	*PLAUR-IRGC*	Stem cell-iPSC	*PLAUR*	0.0148
eQTL	rs8109584	*PLAUR-IRGC*	Brain-Prefrontal Cortex	*ZNF576*	0.0188
eQTL	rs8109584	*PLAUR-IRGC*	Stem cell-iPSC	*ZNF404*	0.0217
eQTL	rs8109584	*PLAUR-IRGC*	Blood	*ETHE1*	0.0261
eQTL	rs8109584	*PLAUR-IRGC*	Kidney	*ZNF45*	0.0325
eQTL	rs8109584	*PLAUR-IRGC*	Stem cell-iPSC	*ZNF224*	0.035
eQTL	rs8109584	*PLAUR-IRGC*	Stem cell-iPSC	*ZNF234*	0.0363
eQTL	rs8109584	*PLAUR-IRGC*	Stem cell-iPSC	*PLAUR*	0.0378
eQTL	rs8109584	*PLAUR-IRGC*	Stem cell-iPSC	*PLAUR*	0.0393
eQTL	rs8109584	*PLAUR-IRGC*	Blood	*ZNF226*	0.0396
eQTL	rs8109584	*PLAUR-IRGC*	Blood	*LYPD5*	0.0397
eQTL	rs8109584	*PLAUR-IRGC*	Kidney	*ZNF428*	0.0416
eQTL	rs8109584	*PLAUR-IRGC*	Blood	*ZNF235*	0.045
eQTL	rs8109584	*PLAUR-IRGC*	Stem cell-iPSC	*ZNF226*	0.0461
eQTL	rs8109584	*PLAUR-IRGC*	Stem cell-iPSC	*ZNF230*	0.0487
eQTL	rs8109584	*PLAUR-IRGC*	Blood	*PINLYP*	0.0488
eQTL	rs12921822	*RBFOX1*	Brain-Prefrontal Cortex	*AC009135.1*	0.00908
hQTL	rs8109584	*PLAUR-IRGC*	Blood-Neutrophils CD16+	*PLAUR*	0.00262
hQTL	rs8109584	*PLAUR-IRGC*	Blood-T cell CD4+ naive	*ZNF575*	0.00385
m6AQTL	rs78631167	*PLAUR-IRGC*	Lymphocyte	*IRGQ*	0.0406
mQTL	rs12639023	*LINC02005-CNTN3*	Blood	*AKR1B1P2*	1.21E-83
mQTL	rs8109584	*PLAUR-IRGC*	Blood-Monocytes CD14+	*PLAUR*	7.99E-11
mQTL	rs8109584	*PLAUR-IRGC*	Blood-Monocytes CD14+	*PLAUR*	3.48E-06

mQTL	rs8109584	*PLAUR-IRGC*	Blood-Monocytes CD14+	*PLAUR*	0.000507
mQTL	rs8109584	*PLAUR-IRGC*	Blood-Monocytes CD14+	*XRCC1*	0.0036
mQTL	rs12921822	*RBFOX1*	Blood	*RBFOX1*	2.43E-18
mQTL	rs12921822	*RBFOX1*	Blood	*RBFOX1*	6.26E-13
mQTL	rs12921822	*RBFOX1*	Blood-Monocytes CD14+	*RBFOX1*	0.00409
tuQTL	rs12639023	*LINC02005-CNTN3*	Stem cell-iPSC	*PDZRN3*	0.00778
tuQTL	rs12639023	*LINC02005-CNTN3*	Stem cell-iPSC	*PDZRN3*	0.0141
tuQTL	rs12639020	*LINC02005-CNTN3*	Stem cell-iPSC	*PDZRN3*	0.00778
tuQTL	rs12639020	*LINC02005-CNTN3*	Stem cell-iPSC	*PDZRN3*	0.0141
tuQTL	rs8109584	*PLAUR-IRGC*	Stem cell-iPSC	*PLAUR*	0.00198
tuQTL	rs8109584	*PLAUR-IRGC*	Stem cell-iPSC	*KCNN4*	0.00532
tuQTL	rs8109584	*PLAUR-IRGC*	Stem cell-iPSC	*ZNF234*	0.00554
tuQTL	rs8109584	*PLAUR-IRGC*	Stem cell-iPSC	*PHLDB3*	0.00641
tuQTL	rs8109584	*PLAUR-IRGC*	Stem cell-iPSC	*ZNF222*	0.0086
tuQTL	rs8109584	*PLAUR-IRGC*	Stem cell-iPSC	*PHLDB3*	0.00884
tuQTL	rs8109584	*PLAUR-IRGC*	Stem cell-iPSC	*KCNN4*	0.00904
tuQTL	rs8109584	*PLAUR-IRGC*	Stem cell-iPSC	*ZNF575*	0.00907
tuQTL	rs8109584	*PLAUR-IRGC*	Stem cell-iPSC	*XRCC1*	0.00961
tuQTL	rs8109584	*PLAUR-IRGC*	Stem cell-iPSC	*KCNN4*	0.00994
tuQTL	rs8109584	*PLAUR-IRGC*	Stem cell-iPSC	*KCNN4*	0.0106
tuQTL	rs8109584	*PLAUR-IRGC*	Stem cell-iPSC	*ZNF155*	0.0107
tuQTL	rs8109584	*PLAUR-IRGC*	Stem cell-iPSC	*ZNF222*	0.0189
tuQTL	rs8109584	*PLAUR-IRGC*	Stem cell-iPSC	*ETHE1*	0.02
tuQTL	rs8109584	*PLAUR-IRGC*	Stem cell-iPSC	*ETHE1*	0.02
tuQTL	rs8109584	*PLAUR-IRGC*	Stem cell-iPSC	*ZNF234*	0.0224
tuQTL	rs8109584	*PLAUR-IRGC*	Stem cell-iPSC	*SMG9*	0.0241
tuQTL	rs8109584	*PLAUR-IRGC*	Stem cell-iPSC	*ZNF155*	0.0243
tuQTL	rs8109584	*PLAUR-IRGC*	Stem cell-iPSC	*ZNF576*	0.0265
tuQTL	rs8109584	*PLAUR-IRGC*	Stem cell-iPSC	*ZNF234*	0.0276
tuQTL	rs8109584	*PLAUR-IRGC*	Stem cell-iPSC	*ZNF234*	0.0276
tuQTL	rs8109584	*PLAUR-IRGC*	Stem cell-iPSC	*PHLDB3*	0.0277
tuQTL	rs8109584	*PLAUR-IRGC*	Stem cell-iPSC	*ZNF222*	0.0289
tuQTL	rs8109584	*PLAUR-IRGC*	Stem cell-iPSC	*ZNF576*	0.0298
tuQTL	rs8109584	*PLAUR-IRGC*	Stem cell-iPSC	*PLAUR*	0.0317
tuQTL	rs8109584	*PLAUR-IRGC*	Stem cell-iPSC	*XRCC1*	0.0341
tuQTL	rs8109584	*PLAUR-IRGC*	Stem cell-iPSC	*XRCC1*	0.0356

tuQTL	rs8109584	*PLAUR-IRGC*	Stem cell-iPSC	*SMG9*	0.0386
tuQTL	rs8109584	*PLAUR-IRGC*	Stem cell-iPSC	*XRCC1*	0.0389
tuQTL	rs8109584	*PLAUR-IRGC*	Stem cell-iPSC	*ZNF222*	0.0392
tuQTL	rs8109584	*PLAUR-IRGC*	Stem cell-iPSC	*ZNF155*	0.0392
tuQTL	rs8109584	*PLAUR-IRGC*	Stem cell-iPSC	*ZNF283*	0.0407
tuQTL	rs8109584	*PLAUR-IRGC*	Stem cell-iPSC	*XRCC1*	0.0424
tuQTL	rs8109584	*PLAUR-IRGC*	Stem cell-iPSC	*ZNF155*	0.0467
tuQTL	rs8109584	*PLAUR-IRGC*	Stem cell-iPSC	*ZNF575*	0.0496

Supplementary Table S3. Allele frequency difference among AMI patients with cardiac death and living patients, and controls

Gene	Chromosome	Position	rsID	Allele frequency		
				Cardiac death group	Alive group	Control
FRG1CP-FRG1DP	20	28,772,995	rs1277393322	0.06818	0.08456	0.01782
MIR1263-LINC01324	3	164,704,630	rs1351282285	0	0.03463	0.08504
MUC4	3	195,788,126	rs1560389462	0	0.001645	0.01577
LINC02005-CNTN3	3	73,919,588	rs12639020	0.3333	0.2659	0.2155
LINC02005-CNTN3	3	73,919,636	rs12639023	0.3977	0.2958	0.2171
RBFOX1	16	7,002,773	rs12921822	0.2391	0.2267	0.2292
PLAUR-IRGC	19	43,676,115	rs78631167	0.02273	0.05545	0.07824
PLAUR-IRGC	19	43,676,102	rs8109584	0.04545	0.04868	0.07732

References

1. Martin M. Cutadapt removes adapter sequences from high-throughput sequencing reads. EMBnet journal. 2011;17(1):10-2.

2. Li H, Durbin R. Fast and accurate short read alignment with Burrows–Wheeler transform. bioinformatics. 2009;25(14):1754-60.

3. Poplin R, Ruano-Rubio V, DePristo MA, Fennell TJ, Carneiro MO, Van der Auwera GA, et al. Scaling accurate genetic variant discovery to tens of thousands of samples. BioRxiv. 2017:201178.

4. Jeon Y, Jeon S, Choi W-H, An K, Choi H, Kim B-C, et al. Genome-wide analyses of early-onset acute myocardial infarction identify 29 novel loci by whole genome sequencing. Human Genetics. 2022:1-13.

5. McLaren W, Gil L, Hunt SE, Riat HS, Ritchie GR, Thormann A, et al. The ensembl variant effect predictor. Genome biology. 2016;17(1):1-14.

6. Wang K, Li M, Hakonarson H. ANNOVAR: functional annotation of genetic variants from high-throughput sequencing data. Nucleic acids research. 2010;38(16):e164-e.
